# Association between pre-operative mental health and recovery outcomes following total knee arthroplasty

**DOI:** 10.1038/s41598-026-44799-5

**Published:** 2026-03-19

**Authors:** Katrin Osmanski-Zenk, Charlotte Brandt, Wolfram Mittelmeier, Martina Rohde-Lindner, Christoph Lutter, Peter Kropp, Annett Klinder

**Affiliations:** 1 Orthopedic Clinic and Policlinic, University Rostock University Medical Center, Doberanerstrasse 142, 18057 Rostock, Germany; 2 Institute of Medical Psychology and Medical Sociology, University Rostock Medical Center, Rostock, Germany

**Keywords:** Depression, Anxiety, PROMs, Joint function, Pain, Quality of life, Quality of life, Human behaviour

## Abstract

This prospective study of 123 patients examined the relationship between mental health and the recovery process in terms of pain, function, and quality of life up to one year after total knee arthroplasty (TKA). Mental health questionnaires such as the Hospital Anxiety and Depression Scale (HADS) and the Beck Depression Inventory Fast Screen (BDI-FS) were completed pre-operatively as well as at three months and 12 months post-operatively. In addition, patient-reported outcome measures such as the Oxford Knee Score (OKS), EuroQol-5 Dimensions (EQ-5D-3 L), and pain intensity were examined to determine the association between functional and psychological symptoms. There were no significant correlations between pre-operative mental health and post-operative outcomes at three months. However, at 12 months follow-up, preoperative anxiety and depression correlated with worse pain, function, and quality of life scores. Group comparisons with groups defined by the cut-off ranges of the psychological health scores also showed these differences. Additionally, poor outcomes for joint function, quality of life, and pain were associated with high depression scores at 12 months post-operatively. This study highlights the importance of pre-operative mental health for the success of TKA, but also the association between poor functional outcomes and mental health post-operatively.

**Supplementary Information:**

The online version contains supplementary material available at 10.1038/s41598-026-44799-5.

## Introduction

Total knee arthroplasty (TKA) represents an effective surgical treatment option for patients with advanced osteoarthritis. However, despite the overall success of the procedure approximately 10 to 20% of patients are dissatisfied with the outcome after TKA^1–3^.

While the functional outcome and patient satisfaction may depend on various factors, mental health was identified as a crucial risk factor when assessing dissatisfaction after TKA^3^. The prevalence of anxiety symptoms and depression, the two most common psychological symptoms, is approximately 20% in osteoarthritis patients^4,5^. While Stubbs et al.^4^ found a high prevalence of depressive and anxiety symptoms in individuals with osteoarthritis, they stated that it is nevertheless not certain whether depression and anxiety are more common in osteoarthritis patients compared to individuals without osteoarthritis. Immediately after surgical treatment of osteoarthritis patients, there is less evidence for an association of psychological assessments with short-term outcomes^6,7^. While pain catastrophizing is a consistent predictor of pain in the first 48 h after surgery, data on the influence of anxiety and depression are inconclusive^7^. Also, the length of hospital stay (LoS) was significantly longer in patients with worse psychological health, but the differences in LoS were often less than a day which limits Klicken oder tippen Sie hier, um Text einzugeben.Klicken oder tippen Sie hier, um Text einzugeben.their clinical significance^6^. Rather, it is thought that mental health plays a significant role for the mid- to long-term satisfaction after TKA and several systematic reviews showed that poor mental health, including anxiety, depression and pain catastrophizing, was associated with worse post-operative outcomes, such as increased pain, reduced function, and lower patient satisfaction^2,3,8–10^. In the most recent review by Nakano et al.^3^, mental health problems were reported more frequently than any other risk factor (in 13 of 181 studies) to affect patient satisfaction. However, in some of the reviewed studies, mental health was assessed only post-operatively, thus not allowing conclusions regarding its predictive potential^11–13^. Additionally, while worse mental health was a good predictor of patient dissatisfaction and overall quality of life, it was not always linked with worse functional outcome and pain in the disease-specific scores after TKA^14,15^. For example, Ali et al.^16^ showed that patients suffering from pre-operative anxiety symptoms and/or depression had an up to six times higher risk of patient dissatisfaction in the four years after TKA. However, in a preceding study^17^, the same authors reported that, apart from higher pain scores and a slightly reduced range of motion, there were no differences in the clinical exams and radiographic analyses between satisfied and dissatisfied patients. Moreover, the dissatisfied patients performed equally well in the functional tests as the satisfied patients. Also, the systematic reviews that focused on mental health as a predictor of functional recovery after TKA showed that up to 50% of the conducted studies failed to confirm an association^9,10^. These include for instance a study with large cohort of over 900 patients by Lingard & Riddl^18^ as well as a more recent study by Wylde et al.^19^ with 266 patients.

The transferability of these diverse results to all outcome measures after TKA in general is further complicated by the use of a variety of questionnaires in the existing studies to assess mental health status, including just using a single question, e.g. from the EQ-5D^9^.

As we generally utilize Patient Reported Outcome Measures (PROMs), including the disease-specific Oxford Knee Score (OKS) and EQ-5D-3 L for general health, to track the recovery progress of our TKA patients after surgery^20^, the aim of our study was not only to assess the prevalence of anxiety and depression in our local patient population, but also to investigate whether there is an association between anxiety and/or depression and the changes between pre-operative (pre-OP) and post-operative PROM scores for up to a year after TKA. For this purpose a prospective, monocentric study was conducted at an Orthopedic Clinic.

## Methods

**Fig. 1:**
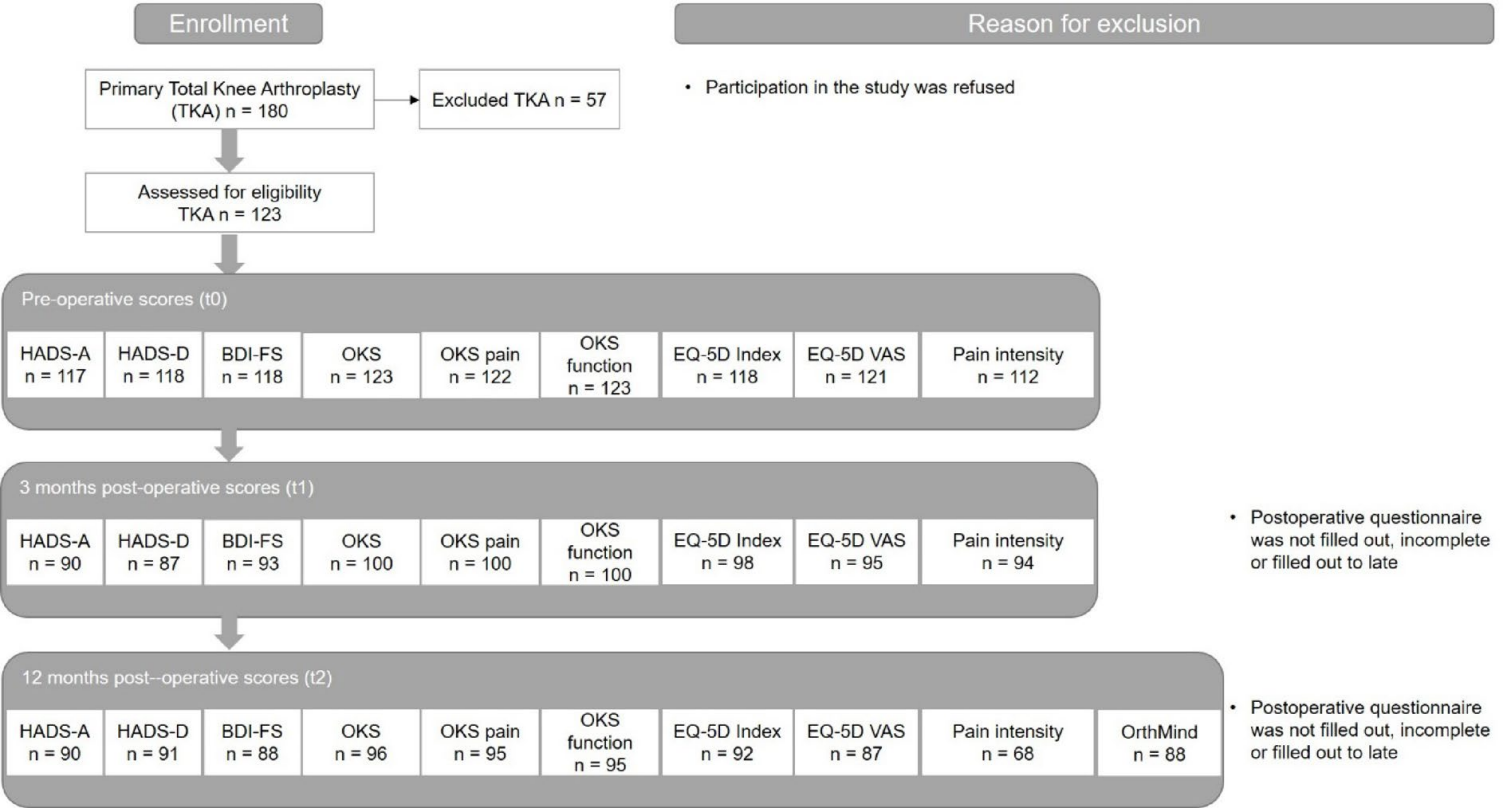
Inclusion criteria, Data of questionnaires used with number of responses. HADS: Hospital Anxiety and Depression Scale; HADS-D: Depression; HADS-A: Anxiety; BDI-FS: Beck Depression Inventory Fast Screen; OKS: Oxford Knee Score; EQ-5D: EuroQol-5 Dimensions; VAS: Visual Analogue Scale; t: timepoint; n: number of patients.

The study was approved by the local ethics committee and a positive ethics vote was obtained (No. of vote: A2019-0057). In total 123 patients who were scheduled for primary TKA between January 2019 and September 2020 in our clinic were recruited for the study (Fig. 1). Patients were informed about the study by a physician on the day when the indication for elective TKA was confirmed, which took place four to six weeks before surgery. The study documents were then provided by the nursing staff. After obtaining written consent from eligible patients, patients completed combined questionnaires PROMs including OKS, EuroQol-5 Dimensions (EQ-5D-3 L) and a Visual Analogue Scale (VAS) for pain rating at home. On the day of admission to hospital, the PROMs were collected by the admission nurse and the pre-operative mental health questionnaires, Hospital Anxiety and Depression Scale (HADS) and Beck Depression Inventory Fast Screen (BDI-FS), were also handed out. These were completed by the patient during the inpatient admission procedure immediately before or when the patients were allotted their rooms and transferred to the surgical ward (t0). The only exception were patients scheduled for surgery on a Monday, who underwent the inpatient admission procedure already on the preceding Friday. For all questionnaires the respective approved German version was used as indicated below and the types and titles of the questionnaires were visible to the patients.

### Hospital anxiety and depression scale (HADS)

The HADS measures the state of mental well-being and is used to screen for anxiety and depression symptoms in the clinical setting^21^. The original HADS questionnaire was developed in 1983^22^ and allows an assessment of mental health despite the simultaneous presence of physical pain. Therefore, the use of the HADS has proven to be useful for the assessment of patients with osteoarthritis of the knee^23,24^. The third edition of the HADS from 2011 was used in the present study^25^. The HADS contains seven questions each for the evaluation of depression and anxiety. Possible answers are ranked on a 4-point Likert scale from 0 (asymptomatic) to 3 points. The respective maximum values for depression (HADS-D) and anxiety (HADS-A) are therefore 21 points. Scores of 0–7 are considered as asymptomatic, 8–10 as doubtful, 11–14 as severe and 15–21 as very severe according to Petermann^26^. The HADS has demonstrated good psychometric properties, with Cronbach’s alpha typically ranging between 0.80 and 0.90 for both subscales^22,26^.

### Beck depression inventory fast screen (BDI-FS)

The BDI-FS measures symptoms of sadness, loss of pleasure, suicidal thoughts, pessimism, past failure, self-dislike and self-criticalness^27^. The BDI-FS consists of seven questions with four response options each. Response options were scored on a 4-point Likert scale from 0 to 3, thus resulting in a possible total score of 0 to 21 points. The cut-off ranges in BDI-FS were 0–3 points for asymptomatic individuals, 4–6 points for mild depression, 7–9 points for moderate depression and 10–21 points for severe depression. The BDI-FS has demonstrated high internal consistency (Cronbach’s alpha = 0.839) and good construct validity^27,28^.

### Oxford knee score (OKS)

The OKS is a validated, joint-specific, patient-reported outcome measure that is used to evaluate pain and functionality after total knee arthroplasty. Interpretation of the OKS was performed according to Murray et al.^29^ with 0 as the worst outcome up to 4 as the best outcome per question (5-point Likert scale) and a maximum value of 48 for all 12 questions of the OKS. The OKS has consistently demonstrated high levels of reliability and validity when used with orthopaedic patient groups, with Cronbach’s alpha values typically exceeding 0.90^30^. Additionally, the OKS total score can be subdivided in a pain and a function score following the approach described by Harris et al.^31^. The pain subscore included items 1, 4, 5, 6, 8, 9, and 10, while the function subscore included items 2, 3, 7, 11 and 12. To enhance interpretability, both subscores were linearly transformed to a 0–100 scale, with 0 indicating the worst and 100 the best possible outcome^31^.

### EuroQol-5 dimensions (EQ-5D-3 L)

The EQ-5D-3 L is a widely used generic instrument for assessing health-related quality of life and includes five dimensions (mobility, self-care, usual activities, pain/discomfort, and anxiety/depression) each with three levels of severity. The EQ-5D index values were calculated using the German value set based on time trade-off (TTO) valuations from the general population, as recommended by the EuroQol Group^32^. Additionally, a visual analogue scale (EQ-VAS) records the respondent’s overall perceived health on a 0–100 (worst to best) scale.

Finally the patients were asked to complete a VAS pain scale ranging from 0 to 10 (0 = no pain, 10 = extreme pain). Demographic data such as age, gender and American Society of Anesthesiologists (ASA) score were obtained from the patients´ medical records. Comorbidities were evaluated with the Charlson Comorbidity Index (CCI)^33^.

Since clinically relevant changes after TKA occur primarily in the first postoperative year^34,35^ the follow-up timepoints for patients in this study were scheduled at three months (t1) and 12 months (t2) after TKA. Post-operative data were collected by post. At the 12 months follow-up, in addition to the previous mentioned questionnaires, the patients received a questionnaire with seven study-specific exploratory questions enquiring about their employment status, pension applications, care level and current pain. This related solely to the four weeks before the 12 months follow-up date.

### Statistical analysis

Statistical analyses were performed with IBM SPSS Statistics 20.0 (IBM Corp., New York, USA) including descriptive statistics for continuous and categorical variables. Normal distribution of data was analyzed using the Shapiro-Wilk test. In the comparisons of the post-operative scores (psychological health measures and PROMs) to the pre-operative scores, normally distributed continuous values were compared using the paired Student´s t test, while for non-normally distributed data the comparisons were performed with the Wilcoxon test as indicated in the tables. The comparisons for pre-operative to three months data and pre-operative to 12 months data were performed separately to allow the analysis of the maximum available number of data. This meant that even when there was no completed questionnaire at three months for a patient a paired analysis between pre-OP and 12 months could be performed and vice versa. When data were classified according to the cut-off ranges of the pre-operative psychological health scores at least one of the defined groups was always not normally distributed. Therefore, differences between these groups were analyzed with the Kruskal-Wallis test followed by the Bonferroni post hoc test. Correlation analyses were performed according to Spearman. The correlation coefficient r was rated according to Cohen for weak ≥ 0.1, intermediate ≥ 0.3, and strong ≥ 0.5 correlations^36^. Only significant correlations were reported. Two-sided p-values < 0.05 were considered as statistically significant.

## Results

### Response rate

The response rate was 75.6% after three months and 74.0% after 12 months for the mental health questionnaires and 81.3% after three months and 78,0% after 12 months for the PROMs (Fig. 1).

### Demographic data of recruited patients

A total of 72 female (58.5%) and 51 male (41.5%) patients were recruited for the study. The patients had a mean age of 69 years (range 47–88 years), an ASA score of 2.4 ± 0.1 and a CCI of 3.85 ± 0.02. When analyzing patients within the different cut-off ranges for depression and anxiety with regard to sex, age, ASA score and CCI, there were no significant differences for any of the demographic data between the classified groups (Table 1).

**Table 1:** Comparisons of demographic data of patients pre-operatively (t0) between groups based on cut-off ranges in pre-operative mental health questionnaires.

Demographic patient data	Classification of patients according to pre-operative BDI-FS into^(A)^			Comparison of groups (*p* value)
	Asymptomatic	Mild depression	Moderate depression	
Sex: Male (%) Female (%)	45 (91.8) 58 (84.1)	4 (8.2) 5 (7.2)	0 (0.0) 5 (7.2)	0.213^§^
Age (mean ± SD)	69.5 ± 8.9	68.2 ± 11.5	67.0 ± 13.5	0.893^#^
ASA: 1 (%) 2 (%) 3 (%)	4 (80.0) 57 (90.5) 42 (84.0)	0 (0.0) 4 (6.3) 5 (10.0)	1 (20.0) 2 (3.2) 2 (4.0)	0.479^§^
CCI (median [range])	4 [1–10]	5 [1–8]	4 [1–9]	0.959^&^

Demographic patient data	Classification of patients according to pre-operative HADS-D into			Comparison of groups (*p* value)
	Asymptomatic	Doubtful	Severe symptoms	
Sex: Male (%) Female (%)	37 (77.1) 51 (72.9)	5 (10.4) 16 (22.9)	6 (12.5) 3 (4.3)	0.080^§^
Age (mean ± SD)	69.2 ± 9.3	68.1 ± 10.2	68.9 ± 5.4	0.887^#^
ASA: 1 (%) 2 (%) 3 (%)	3 (60.0) 52 (82.5) 33 (66.0)	2 (40.0) 9 (14.3) 10 (20.0)	0 (0.0) 2 (3.2) 7 (14.0)	0.099^§^
CCI (median [range])	3.5 [1–10]	4 [1–9]	3 [2–8]	0.834^&^

Demographic patient data	Classification of patients according to pre-operative HADS-A into			Comparison of groups (*p* value)
	Asymptomatic	Doubtful	Severe symptoms	
Sex: Male (%) Female (%)	44 (88.0) 49 (73.1)	4 (8.0) 10 (14.9)	2 (4.0) 8 (11.9)	0.132^§^
Age (mean ± SD)	69.0 ± 9.1	69.9 ± 10.7	68.0 ± 9.3	0.880^#^
ASA: 1 (%) 2 (%) 3 (%)	4 (80.0) 50 (79.4) 39 (79.6)	1 (20.0) 8 (12.7) 5 (10.2)	0 (0.0) 5 (7.9) 5 (10.2)	0.904^§^
CCI (median [range])	3 [1–10]	4 [1–10]	3.5 [1–8]	0.678^&^

HADS: Hospital Anxiety and Depression Scale; HADS-D: Depression; HADS-A: Anxiety; BDI-FS: Beck Depression Inventory Fast Screen; ASA: American Society of Anesthesiologists score; CCI: Charlson Comorbidity Index.

Comparison between the groups was performed using the indicated statistical tests. Categorical variables were tested with the Chi-Square test (§). Continuous variables were either tested with the ANOVA (#) for normally distributed data or the Kruskal-Wallis test (&) for not normally distributed data. Overall p-values are listed in the last column. As none of the comparisons were significant, no post hoc test were performed. (A) – There was only one female patient with severe depression at t0 according to BDI-FS. This patient was 66 years old with an ASA of 3 and a CCI of 3. While this patient was not included in Table 1, it was considered for the statistical analysis.

### Psychological health scores

**Fig. 2:**
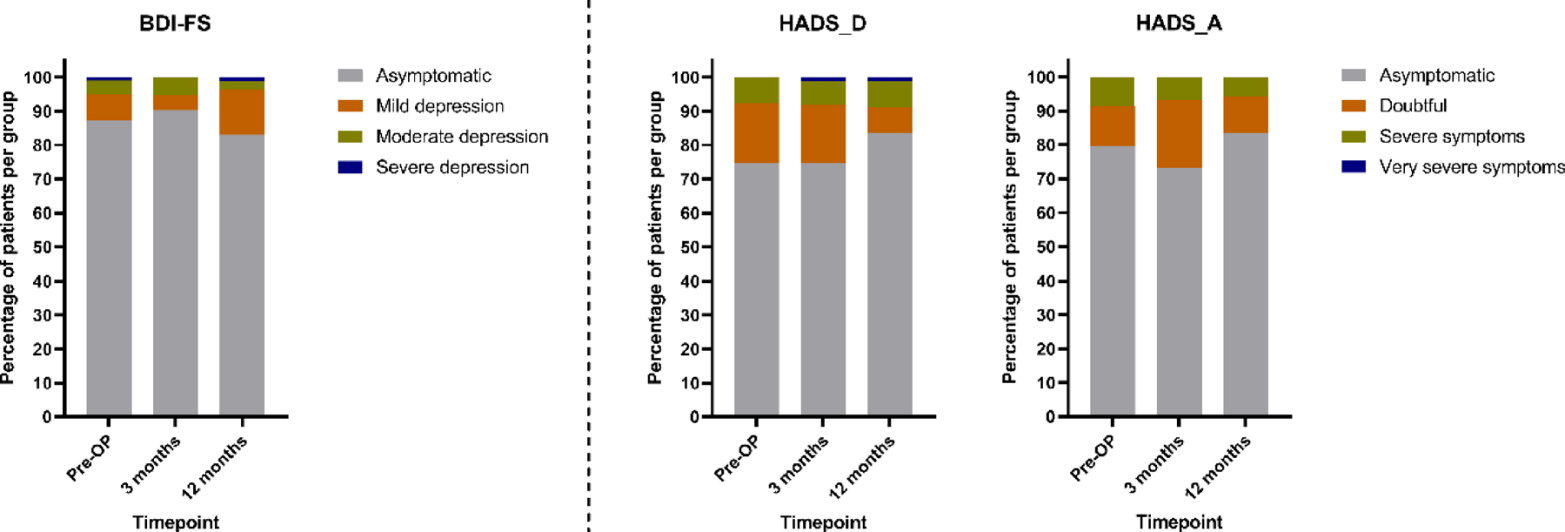
Proportion of patients within the identified cut-off ranges for depression and anxiety in BDI-FS and HADS at the three assessed timepoints (Pre-OP = prior to total knee arthroplasty (TKA), three months after TKA and 12 months after TKA; HADS: Hospital Anxiety and Depression Scale; HADS-D: Depression; HADS-A: Anxiety; BDI-FS: Beck Depression Inventory Fast Screen; Pre-OP: preoperative.

The majority of elective TKA patients did not show signs of anxiety or depression and the percentage of patients within the respective cut-off ranges remained relatively constant over time (Fig. 2).

This was also reflected by the comparison between the pre-operative score values and the scores recorded at three and 12 months after surgery. There were no significant changes regarding depression, neither in BDI-FS nor in HADS-D (Table 2). The reliability of the data was confirmed since depression scores of HADS-D and BDI-FS correlated significantly with each other at all timepoints (pre-operative: *r* = 0.675, three months: *r* = 0.755 and 12 months: *r* = 0.707 with *p* < 0.001 for all three Spearman`s correlation analyses). In contrast to depression, a significant post-operative improvement was observed in anxiety of the TKA patients as assessed by HADS-A (Table 2).

**Table 2:** Comparison of depression and anxiety scores of three and 12 months after total knee arthroplasty to pre-operative values.

Psychometric questionnaire	Timepoint	*N*	Score Mean ± SD	Score Median [range]	Comparison to pre-OP (*p* value)
BDI-FS	Pre-OP	118	1.51 ± 2.11	1 [0–10]	
	3 months	93	1.55 ± 1.94	1 [0–9]	n.s.
	12 months	88	1.50 ± 2.06	1 [0–10]	n.s.
HADS-D	Pre-OP	118	5.02 ± 3.38	4 [0–13]	
	3 months	87	4.82 ± 3.68	4 [0–18]	n.s.
	12 months	91	4.27 ± 3.80	3 [0–19]	n.s.
HADS-A	Pre-OP	117	5.46 ± 3.24	5 [0–14]	
	3 months	90	4.90 ± 3.35	4 [0–12]	0.01^#^
	12 months	90	4.14 ± 3.52	3 [0–14]	< 0.001^#^

BDI-FS: Beck Depression Inventory Fast Screen; HADS: Hospital Anxiety and Depression Scale, HADS-D: Depression, HADS-A: Anxiety; N: number of patients; SD: Standard Deviation; # = Wilcoxon Test; n.s.= not significant.

### Patient reported outcome measures

Concurrent to the post-operative improvement in anxiety there was also increased patient outcome at three months and 12 months after surgery as reflected by the significantly improved scores in OKS including both subscores, reduced pain and enhanced quality of life (Table 3).

**Table 3:** Comparison of patient reported outcome measures scores of three and 12 months after total knee arthroplasty to pre-operative values.

PROM	Timepoint	*N*	Score Mean ± SD	Score Median [range]	Comparison to pre-OP (*p* value)
OKS	Pre-OP	123	20.44 ± 7.9	21 [2–37]	
	3 months	100	30.21 ± 7.73	31 [14–46]	< 0.001^§^.
	12 months	96	33.53 ± 10.35	36 [0–48]	< 0.001^#^
OKS function	Pre-OP	123	46.75 ± 18.19	45 [0–85]	
	3 months	100	62.03 ± 17.96	60 [30–100]	< 0.001^§^
	12 months	95	68.62 ± 20.26	70 [25–100]	< 0.001^#^
OKS pain	Pre-OP	122	39.96 ± 16.68	42–84 [3.57– 82.11]	
	3 months	100	63.62 ± 17.19	64.26 [21.42–96.39]	< 0.001^§^.
	12 months	95	71.98 ± 21.55	78.54 [10.71–100]	< 0.001^#^
EQ-5D	Pre-OP	118	0.58 ± 0.28	0.79 [-0.14–1.00]	
	3 months	98	0.84 ± 0.15	0.89 [0.26–1.00]	< 0.001^#^
	12 months	92	0.85 ± 0.21	0.89 [0.18–1.00]	< 0.001^#^
EQ-5D VAS	Pre-OP	121	50.67 ± 24.18	50 [0–100]	
	3 months	95	70.76 ± 19.43	75 [5–100]	< 0.001^#^
	12 months	87	68.67 ± 21.21	70 [7–100]	< 0.001^#^
VAS pain	Pre-OP	112	7.96 ± 1.56	8 [5–10]	
	3 months	94	3.95 ± 2.10	4 [0–9]	< 0.001^#^
	12 months	68	4.40 ± 2.47	4 [0–10]	< 0.001^§^

OKS: Oxford Knee Score; EQ-5D: EuroQuol-5 Dimensions; VAS: visual analog scale; N: number of patients; § = paired Student`s t test; # = Wilcoxon Test.

### Association between pre-operative mental health status and post-operative patient satisfaction

Correlation analyses of the entire patient cohort revealed no association of pre-operative mental health status with outcome measures after three months and only weak correlations to selected PROMs after 12 months (Table 4). In particular, higher depression scores in BDI-FS as well as HADS-D prior to surgery seemed to be linked to continued pain at 12 months after TKA. However, there were also associations between pre-operative anxiety and post-operative pain as well as general well-being (EQ-5D3L VAS) at 12 months after TKA.

**Table 4:** Significant correlations according to Spearman between pre-operative (t0) psychological health scores and patient reported outcome measures scores 12 months after total knee arthroplasty (t2).

Psychological health questionnaire at t0	PROMs at t2	*N*	Correlation coefficient *r*	*p* value
BDI-FS	OKS	93	-0.209	0.044
BDI-FS	OKS pain	92	-0.233	0.025
BDI-FS	VAS pain	65	0.318	0.010
BDI-FS	EQ-5D VAS	84	-0.225	0.039
HADS-D	OKS	92	-0.250	0.016
HADS-D	OKS pain	91	-0.266	0.011
HADS-D	VAS pain	66	0.257	0.037
HADS-A	VAS pain	66	0.331	0.007
HADS-A	EQ-5D VAS	82	-0.256	0.021

HADS: Hospital Anxiety and Depression Scale; HADS-D: Depression; HADS-A: Anxiety; BDI-FS: Beck Depression Inventory Fast Screen; OKS: Oxford Knee Score; EQ-5D: EuroQuol-5 Dimensions; VAS: visual analog scale; N: number of patients.

There were also no significant correlations between the pre-operative psychological health scores and the daily life activities assessed with the seven study-specific exploratory questions (*N* = 88) at 12 months after TKA.

However, when patients were classified according to the cut-off ranges of the different mental health questionnaires based on the pre-operative scores, it was observed that patients with pronounced depression prior to surgery (mild depression according to BDI-FS, severe symptoms according to HADS-D at t0) reported worse results in almost all PROMs, including the functional outcome, at 12 months after TKA (Table 5, Kruskal-Wallis test: *p* < 0.05). There were no significant differences at 12 months when classifying patients based on anxiety scores. Also no differences in PROMs were detected at three months after TKA between patient groups that were classified according to the pre-operative mental health scores.

**Table 5:** Comparisons of patient reported outcome measures at 12 month after total knee arthroplasty (t2) between groups based on cut-off ranges in pre-operative mental health questionnaires.

PROM score	Classification of patients according to pre-operative BDI-FS into			Comparison of groups^$^ (*p* value)
Median [range] at t2	Asymptomatic	Mild depression	Moderate depression	
OKS pain	78.54 ^a^ [28.56–100]	48.98 ^a^ [10.71–82.11]	46.41 [28.56–67.83]	0.007
VAS pain	4 ^a, b^ [0–10]	7 ^a^ [2–10]	8 ^b^ [5–10]	0.013
EQ-5D VAS	75 ^a^ [9–100]	50 ^a^ [7–80]	50 [35–70]	0.007

PROM score	Classification of patients according to pre-operative HADS-D into			Comparison of groups (*p* value)
Median [range] at t2	Asymptomatic	Doubtful	Severe symptoms	
OKS	37 ^a^ [16–48]	35 [0–46]	18 ^a^ [8–36]	0.011
OKS function	75 ^a^ [30–100]	75 ^b^ [30–95]	37.5 ^a, b^ [25–70]	0.012
OKS pain	78.54 ^a^ [35.70–100]	74.97 [28.56–96.39]	37.49 ^a^ [10.71–78.54]	0.013
VAS pain	4 ^a^ [0–10]	6.5 [0–9]	7 ^a^ [3–10]	0.027
EQ-5D	0.89 ^a^ [0.18–1.00]	0.89 ^b^ [0.18–1.00]	0.48 ^a, b^ [0.18–0.89]	0.004

$ = comparison between the groups was performed with Kruskal-Wallis test including Bonferroni´s post hoc test: overall p-values are listed in the last column, while results of the post hoc test are displayed as superscript letters. The same superscript letters in one row indicate that the thus labeled scores differ significantly from each other according to Bonferroni´s post hoc test. Only significant differences were reported.

HADS: Hospital Anxiety and Depression Scale; HADS-D: Depression; HADS-A: Anxiety; BDI-FS: Beck Depression Inventory Fast Screen; OKS: Oxford Knee Score; EQ-5D: EuroQuol-5 Dimensions; VAS: visual analog scale.

Patients, who were classified as mild and moderate depression in BDI-FS or with severe symptoms in HADS-D prior to surgery, did not only show poorer outcome measures at 12 months after TKA, but also their improvement from pre-operative scores was significantly lower compared to asymptomatic patients (Table 6, Kruskal-Wallis test: *p* < 0.05).

**Table 6:** Comparisons of the changes in patient reported outcome measures (∆ = difference of t2-t0) between groups based on cut-off ranges in pre-operative mental health questionnaires.

Change in PROM score	Classification of patients according to pre-operative BDI-FS into			Comparison of groups^$^ (*p* value)
Median of t2-t0 [range]	Asymptomatic	Mild depression	Moderate depression	
∆VAS pain	-4 [(-10) – 2]	0 [(-3) – 4]	-0.5 [(-1) – 0]	0.010

Change in PROM score	Classification of patients according to pre-operative HADS-D into			Comparison of groups (*p* value)
Median of t2-t0 [range]	Asymptomatic	Doubtful	Severe symptoms	
∆OKS	15 ^a^ [(-17) – 30]	12.64 [(-20) – 42]	0.5 ^a^ [(-27) – 16]	0.032
∆OKS function	20 ^a^ [(-35) – 70]	20 ^b^ [(-25) – 80]	-5 ^a, b^ [(-55) – 15]	0.014
∆VAS pain	-4 ^a^ [(-10) – 2]	-1.5 [(-9) – 0]	0 ^a^ [(-5) – 4]	0.012
∆EQ-5D	0.21 ^a^ [(-0.72) – 0.74]	0.30 ^b^ [(-0.09) – 0.83]	-0.08 ^a, b^ [(-0.61) – 0.10]	0.004

$ = comparison between the groups was performed with Kruskal-Wallis test including Bonferroni´s post hoc test: overall p-values are listed in the last column, while results of the post hoc test are displayed as superscript letters. The same superscript letters in one row indicate that the thus labeled scores differ significantly from each other according to Bonferroni´s post hoc test. Only significant differences were reported.

HADS: Hospital Anxiety and Depression Scale; HADS-D: Depression; HADS-A: Anxiety; BDI-FS: Beck Depression Inventory Fast Screen; OKS: Oxford Knee Score; EQ-5D: EuroQuol-5 Dimensions; VAS: visual analog scale.

### Association between treatment outcome after TKA and post-operative mental health status

Significant correlations were observed for all recorded PROMs at 12 months with the post-operative mental health scores at 12 months after TKA (Table 7). Post-operative associations were found for functional outcomes and pain with depression but also with anxiety. The strongest associations between PROMs and mental health status at 12 months were determined for HADS-D with all the PROMs (*r* > -0.5 or *r* > 0.5).

**Table 7:** Significant correlations according to Spearman at 12 months after total knee arthroplasty (t2) between post-operative mental health scores and patient reported outcome measures.

Psychological health questionnaire at t2	PROMs at t2	*N*	Correlation coefficient *r*	*p* value
BDI-FS	OKS	86	-0.404	< 0.001
BDI-FS	OKS function	86	-0.403	< 0.001
BDI-FS	OKS pain	86	-0.380	< 0.001
BDI-FS	VAS pain	63	0.349	0.005
BDI-FS	EQ-5D	84	-0.461	< 0.001
BDI-FS	EQ-5D VAS	82	-0.487	< 0.001
HADS-D	OKS	90	-0.599	< 0.001
HADS-D	OKS function	90	-0.577	< 0.001
HADS-D	OKS pain	90	-0.580	< 0.001
HADS-D	VAS pain	65	0.579	< 0.001
HADS-D	EQ-5D	88	-0.558	< 0.001
HADS-D	EQ-5D VAS	82	-0.528	< 0.001
HADS-A	OKS	89	-0.442	< 0.001
HADS-A	OKS function	89	-0.437	< 0.001
HADS-A	OKS pain	89	-0.417	< 0.001
HADS-A	VAS pain	64	0.416	0.001
HADS-A	EQ-5D	87	-0.410	< 0.001
HADS-A	EQ-5D VAS	81	-0.437	< 0.001

HADS: Hospital Anxiety and Depression Scale; HADS-D: Depression; HADS-A: Anxiety; BDI-FS: Beck Depression Inventory Fast Screen; OKS: Oxford Knee Score; EQ-5D: EuroQuol-5 Dimensions; VAS: visual analog scale; N: number of patients.

**Fig. 3:**
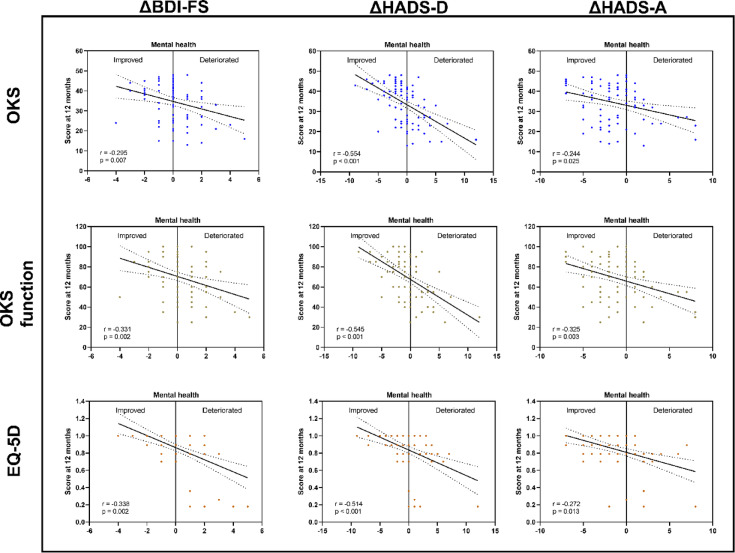
Correlation analyses between the changes (∆) in mental health score with PROMs at 12 months after total knee arthroplasty. In order to calculate improvement or deterioration in mental health for the individual patient, scores for BDI-FS, HADS-D and HADS-A at 12 months were subtracted from the respective pre-operative scores. The resulting changes were labeled ∆BDI-FS, ∆HADS-D, and ∆HADS-A; HADS: Hospital Anxiety and Depression Scale; HADS-D: Depression; HADS-A: Anxiety; BDI-FS: Beck Depression Inventory Fast Screen; OKS: Oxford Knee Score; EQ-5D-3 L: EuroQuol-5 Dimensions; PROM: patient reported outcome measures.

This was supported when analyzing how the psychological health scores of the patients changed over the follow-up period. Figure 3 shows that improved mental health scores (negative values due to calculating the difference as t2-t0) were associated with higher outcome measures at 12 months in the PROMs as depicted for OKS (Fig. 3), OKS function (Fig. 3) and EQ-5D-3 L (Fig. 3).

Additionally to the graphically displayed correlations in Fig. 3, there were further significant correlations of EQ-5D VAS with ∆BDI-FS and ∆HADS-D (*r* = -0.307 and *r* = -0.372, respectively), OKS pain with ∆BDI-FS and ∆HADS-D (*r* = -0.223 and *r* = -0.509 respectively) and VAS pain with ∆HADS-D (*r* = 0.452).

## Discussion

Although similar studies existed prior to this study, our analysis contributes to the growing body of evidence by applying established instruments in a standardized clinical setting with a focus on temporal changes in patient-reported outcomes. Our results showed that while mental health status prior to surgery was not associated with joint function and pain at three months after TKA, significant associations with outcome measures were observed at 12 months postoperatively. The relationship was especially apparent when patients were classified according to the cut-off values into asymptomatic and symptomatic patients, revealing that patients with depressive symptoms showed less improvement compared to asymptomatic patients. In line with our study objective, these findings indicate that pre-operative mental health status is associated with mid-term but not early post-operative patient-reported outcomes after TKA.

Based on the prevalence of depression and anxiety, our cohort seems to differ from previous studies. While it was reported that the prevalence of these symptoms is relatively high (20%) in patients with osteoarthritis^3,4^, the percentage of depression with 8–13% and anxiety with 9% was slightly lower in our osteoarthritis patient population prior to surgery. It was also lower than data reported in previous TKA studies^4^, but higher than the general prevalence among older adults in Germany (5%) which is particularly low compared to similar age groups of the general public in other countries such as the UK (9%) or the US (20%)^37^. In contrast to a study in an elderly German population^38^, we also observed no differences regarding age or the number of comorbidities between asymptomatic and symptomatic patients.

But despite these differences and the relatively small number of symptomatic patients our results are in accordance with several systematic reviews and meta-analyses^7,9,39^ which identified psychological variables, especially anxiety, catastrophizing, and depression, as consistent predictors of suboptimal postoperative outcomes, including pain and function. In general, our study confirmed that mental health correlated well with function and quality of life at the respective timepoints (Supplementary Table S1). Similar to the results here, numerous studies demonstrated that psychological factors, such as depression, anxiety, and psychological distress, are associated with less favorable outcomes following total knee arthroplasty (TKA). Both prospective and retrospective studies consistently reported that patients with lower preoperative mental health scores exhibited reduced improvements in pain, function, and quality of life after surgery^40–42^. These associations extend beyond clinical outcomes, as reflected in lower patient satisfaction. Recent evidence further substantiated this relationship, e.g. Aalders et al.^43^ found that both anxiety and depression prior to TKA were independently associated with worse pain and subjective function one year postoperatively. Similarly, Scattergood et al.^44^ identified depressive symptoms as one of the strongest predictors of poorer functional outcomes at one year. Götz et al.^45^, in a large cohort of over 5,000 patients, confirmed that anxiety and depression were significantly associated with lower quality of life one year after surgery, particularly among TKA patients. Moreover, several investigations suggest that the effects of preoperative psychological burden may persist beyond the early postoperative period.

When evaluating our results in more detail there was a difference between patients with depression and patients with anxiety. While pre-operative depressive symptoms were associated with less favorable recovery patterns at 12 months, there was no association with anxiety. In a systematic review by Bletterman et al.^10^ only 50–80% o the studies, that assessed depression and/or anxiety as the psychological measure, reported a link between pre-operative mental health and functional outcome after TKA. In this review of 26 studies, the authors concluded that overall there was no longitudinal association between pre-operative psychological factors and post-operative functional recovery after TKA. Also other studies failed to establish significant links between pre-operative mental health status and post-operative function^15,39,46–50^.

While this could be due to the use of different questionnaires or scores to assess mental health status prior to surgery^9^, the results from our study suggest that the timing of the post-operative assessment could be a crucial factor for establishing an association. We observed that while the psychological health scores correlated significantly with each other at three and 12 months after TKA, a significant association between pre-operative depression scores and outcome measures as assessed by PROMs was only observed at 12 months after surgery, but not at three months. This may be explained by the fact that clinical outcomes are not yet fully consolidated at three months, as functional recovery and patient-reported outcomes often continue to improve up to 12 months postoperatively^34,48^. Similarly, Duivenvoorden et al.^15^ observed that 12 months after surgery functional outcomes after TKA was stronger related to depression. While they also assessed TKA patients at three months and showed improved depression and anxiety score in their patients, no data regarding the association between were presented for the three months timepoint. In contrast, Zhang et al.^51^. observed significant differences already at early timepoints after total joint arthroplasty (TJA) between an asymptomatic group and a group that showed pre-operative symptoms of depression and anxiety according to HADS. Three and six months after TJA, patients in the asymptomatic group achieved significantly better rehabilitation outcomes than patients from the symptomatic group. Zhang et al. concluded that patients without notable HADS scores recovered faster and more successful in the same period after TJA than patients with obvious HADS scores^51^. In the study by Melnic et al.^42^ all patients experienced improved physical function up to approximately six months after surgery. The improvement occurred independent from the pre-operative mental health scores. However, after this initial improvement, those with the poorest mental health scores experienced a steep decline in physical function approximately one year after surgery, from which they did not appear to recover. This underlines that time of the assessment is critical for the results of these studies. An important question concerns the causality of the observed associations in these studies and it is difficult to determine whether a good mental health status leads to better outcomes or whether achieving better joint function and quality of life after surgery positively impacts on mental health. It was shown that pre-operative anxiety and depression can be caused by chronic joint pain and the resulting loss of function in osteoarthritis patients^15,24,47^. Hence, in the long run successful surgical treatment – restored joint function and absence of pain – should lead to improved mental health and several studies described a reduction of anxiety and depression after TKA^46,47,51^. While the anxiety scores significantly improved in our study over the follow-up period in accordance to the previously reported data, this was not observed for the depression scores. However, the patients whose mental health status, for depression as well as for anxiety, improved over time, usually also reported better recovery after TKA in our study. Taking into account the results of our study and those mentioned in the discussion, it can be concluded that both physical and mental health improved following total knee arthroplasty. Most patients experienced gains in pain, function and quality of life at 12 months. The relationship between physical and mental health is mostly bidirectional. Poorer preoperative mental health was consistently associated with less improvement in physical function. Conversely, substantial physical recovery was associated with significant improvements in mental health, particularly among patients with preoperative psychological distress. Thus, success or failure of TKA in an individual patient might considerably shape post-operative mental health trajectories and thus obscure the association of mental health status prior to surgery when analyzing the study population as an entity. Sorel et al.^39^ suggested that patients with severe symptoms may benefit from psychological support to improve the outcome and quality of life. However, receiver operating characteristic (ROC) analyses of our study data were unable to determine threshold mental health scores to identify patients that are prone to treatment failure and thus, might benefit from pre-operative intervention (data not shown).

## Strengths and limitation

By including both the Hospital Anxiety and Depression Scale (HADS) and the Beck Depression Inventory (BDI), our study combined screening and diagnostic-oriented measures, thus offering a comprehensive assessment of psychological distress. Our study directly compared symptomatic and asymptomatic patients, highlighting that differences in PROMs emerge over time and may be linked to psychological adjustment rather than early functional recovery. Furthermore, the study is embedded in routine management of patients in our hospital ensuring standardized surgical technique and a high follow-up rate, thus enhancing its external validity.

We acknowledge that the exclusion of patients who declined to participate may introduce selection bias or self-selection sampling. As we did not perform a statistical analysis comparing participants and non-participants, this remains a limitation of our study.

The use of the German EQ-5D-3 L value set ensures contextual relevance but may affect comparability with studies using different national value sets. These aspects should be considered when interpreting and generalizing the findings to other populations or healthcare settings.

Although there was some attrition between the preoperative and three-month follow-up assessments, the overall follow-up rate remained acceptable and the study still provides valuable insights into postoperative recovery trajectories. Missing data were not imputed, and analyses were based on available cases only. This may introduce bias if missing values are not completely random and should be considered when interpreting the results.

Comorbidities and potential differences in the CCI, which may affect postoperative outcomes, were not controlled for in the present analysis. However, there were no differences in the distribution of ASA scores and CCI between asymptomatic and symptomatic patients., Still, these factors should be considered in future studies.

Although some comparative studies have combined TKA with other forms of total joint arthroplasty, recovery trajectories and functional demands differ between joints; therefore, our findings are intentionally restricted to TKA and should not be extrapolated to THA or other arthroplasty procedures.

## Conclusions and outlook

Our study partly confirmed that pre-operative mental health status is associated with post-operative outcome. Specifically, patients with symptoms of depression prior to surgery showed less improvement in pain and joint function compared to asymptomatic patients at 12 months post-TKA. However, our data also showed that more favorable outcomes were strongly associated with improvements in mental health, whereas less favorable outcomes were related to a deterioration in mental health. Our findings highlight the potential benefit of preoperative psychological screening and timely therapeutic support, including active patient involvement in care, to improve outcomes after TKA. Additionally, tailored follow-up strategies and interdisciplinary collaboration may help optimize recovery and enhance patient-reported outcomes over the long term.

Future directions of our research include longitudinal studies with larger, more diverse patient cohorts to validate and expand our findings. Moreover, we aim to investigate the effectiveness of targeted psychological interventions pre- and postoperatively to improve patient-reported outcomes and overall recovery after TKA. Integration of interdisciplinary care models will also be explored to optimize long-term patient outcomes.

## Supplementary Information

Below is the link to the electronic supplementary material.

Supplementary Material 1

## Data Availability

The data supporting the findings of this study are available upon request from the corresponding author.
