# Supplementary material for: Association between pre-operative mental health and recovery outcomes following total knee arthroplasty
Source: Sci Rep. 2026 Mar 19;16:9541. doi: 10.1038/s41598-026-44799-5 (PMC13004832; doi:10.1038/s41598-026-44799-5)
Supplement: Supplementary file 1 — Supplementary Material 1 [file 41598_2026_44799_MOESM1_ESM.docx]

Supplementary Table S1: Significant correlations between pre-operative (t0) psychological health scores and pre-operative (t=0) patient reported outcome measures scores and between three month post-operative (t1) psychological health scores and patient reported outcome measures three month after total knee arthroplasty (t1)

|  |  | at t0 | | | at t1 | | |  |
| --- | --- | --- | --- | --- | --- | --- | --- | --- |
| **Psychometric questionnaire** | **PROMs** | **N** | **Correlation coefficient r** | **p value** | **N** | **Correlation coefficient r** | **p value** | |
|  |  |  |  |  |  |  |  |  |
| BDI-FS | OKS | 118 | -0.266 | 0.004 | 93 | -0.269 | 0.009 | |
| BDI-FS | OKS function | 118 | -0.212 | 0.021 | 93 | -0.242 | 0.019 | |
| BDI-FS | OKS pain | 117 | -0.277 | 0.002 | 93 | -0.257 | 0.013 | |
| BDI-FS | VAS pain | n.s. | n.s. | n.s. | n.s. | n.s. | n.s. | |
| BDI-FS | EQ-5D | 113 | -0.239 | 0.011 | 91 | -0.228 | 0.029 | |
| BDI-FS | EQ-5D VAS | n.s. | n.s. | n.s. | 88 | -0.293 | 0.006 | |
| HADS-D | OKS | 118 | -0.243 | 0.008 | 86 | -0.285 | 0.008 | |
| HADS-D | OKS function | 118 | -0.215 | 0.019 | 86 | -0.337 | 0.002 | |
| HADS-D | OKS pain | 117 | -0.225 | 0.015 | 86 | -0.216 | 0.046 | |
| HADS-D | VAS pain | n.s. | n.s. | n.s. | n.s. | n.s. | n.s. | |
| HADS-D | EQ-5D | 113 | -0.246 | 0.009 | 84 | -0.359 | 0.001 | |
| HADS-D | EQ-5D VAS | n.s. | n.s. | n.s. | 82 | -0.344 | 0.002 | |
| HADS-A | OKS | 117 | -0.235 | 0.011 | 89 | -0.286 | 0.007 | |
| HADS-A | OKS function | n.s. | n.s. | n.s. | 89 | -0.267 | 0.011 | |
| HADS-A | OKS pain | 116 | -0.259 | 0.005 | 89 | -0.276 | 0.009 | |
| HADS-A | VAS pain | n.s. | n.s. | n.s. | n.s. | n.s. | n.s. | |
| HADS-A | EQ-5D | 112 | -0.194 | 0.041 | n.s. | n.s. | n.s. | |
| HADS-A | EQ-5D VAS | n.s. | n.s. | n.s. | 85 | -0.249 | 0.022 | |

HADS: Hospital Anxiety and Depression Scale; HADS-D: Depression; HADS-A: Anxiety; BDI-FS: Beck Depression Inventory Fast Screen; OKS: Oxford Knee Score; EQ-5D: EuroQuol-5 Dimensions; VAS: visual analog scale; N: number of patients; n.s. = no significant correlation

The appendix is for possible web publication and also for peer-review purposes.
